# Robust circadian clock oscillation and osmotic rhythms in inner medulla reflecting cortico-medullary osmotic gradient rhythm in rodent kidney

**DOI:** 10.1038/s41598-017-07767-8

**Published:** 2017-08-04

**Authors:** Masayuki Hara, Yoichi Minami, Munehiro Ohashi, Yoshiki Tsuchiya, Tetsuro Kusaba, Keiichi Tamagaki, Nobuya Koike, Yasuhiro Umemura, Hitoshi Inokawa, Kazuhiro Yagita

**Affiliations:** 1 Department of Physiology and Systems Bioscience, Kyoto Prefectural University of Medicine, Kyoto, Japan; 2 Department of Nephrology, Graduate School of Medical Science, Kyoto Prefectural University of Medicine, Kyoto, Japan

## Abstract

Circadian clocks in mammals function in most organs and tissues throughout the body. Various renal functions such as the glomerular filtration and excretion of electrolytes exhibit circadian rhythms. Although it has been reported that the expression of the clock genes composing molecular oscillators show apparent daily rhythms in rodent kidneys, functional variations of regional clocks are not yet fully understood. In this study, using macroscopic bioluminescence imaging method of the PER2::Luciferase knock-in mouse kidney, we reveal that strong and robust circadian clock oscillation is observed in the medulla. In addition, the osmotic pressure in the inner medulla shows apparent daily fluctuation, but not in the cortex. Quantitative-PCR analysis of the genes contributing to the generation of high osmotic pressure or the water re-absorption in the inner medulla, such as vasopressin receptors (*V1aR*, *V2R*), urea transporter (*UT-A2*) and water channel (*Aqp2*) show diurnal variations as well as clock genes. Deficiency of an essential clock gene *Bmal1* impairs day-night variations of osmotic pressure gradient in the inner medulla, suggesting that circadian clocks in the medulla part of the kidney may regulate the circadian rhythm of cortico-medullary osmotic pressure gradient, and may contribute physiological day-night rhythm of urination.

## Introduction

In mammals, various physiological aspects, not only sleep/awake cycles, but also energy metabolisms, cardiovascular functions and renal functions exhibit the apparent daily fluctuation^1–4^. It has been elucidated that an intrinsic time keeping system called the circadian clock regulates these physiological rhythms^5^. At the molecular level, the circadian clock is based on transcriptional/translational feedback loops composed of a set of clock genes such as *Period* genes (*Per1, Per2*), *Cryptochrome* (*Cry1, Cry2*), *RevErb* (*RevErba, RevErbb*), *Bmal1* and *Clock* ^1^. In short, BMAL1 and CLOCK heterodimerize and transactivates E-box regulated genes such as *Per1-2*, *Cry1*, and *RevErbs*, whereas translated PERs and CRYs inhibits transcription via E-box^1, 6, 7^. *RevErb* genes inhibit *Bmal1* expression via ROR response element^8^. Although oscillatory peak times are varied, clock genes expressions show clear circadian rhythms^1, 9^. Molecular clock passes rhythmic expression patterns to the clock-controlled output genes (CCGs) that link the oscillator to clock controlled physiological processes via transcriptional regulations^9^. Although the center of mammalian circadian rhythm is in the suprachiasmatic nucleus (SCN), most organs or tissues, and even cultured fibroblasts, have their own circadian clock in each cell level^10–13^.

The kidney is a highly differentiated organ composed from specialized regions, including the glomerular, tubular interstitial, and vascular components^14^. The important renal function is the maintenance of body fluid homeostasis via urine concentrations. Cortico-medullary osmotic pressure gradient in the kidney is achieved by a countercurrent multiplier system and a countercurrent exchanger system, and it contributes to water re-absorption via water channels^15, 16^. Sodium (Na), potassium (K), and urea are important substances for generating appropriate osmotic pressure in the kidney^17^. Urea concentrations show dynamic increase from the outer medulla (OM) to inner medulla (IM), and high osmotic pressure via urea accumulation drives passive re-absorption of Na and chloride (Cl) in the IM^18, 19^.

Day-night variations of urine volume, renal blood flows, and glomerular filtration rate (GFR) have been reported^20–24^. Not only renal excretion of urea and water, but also electrolyte levels in urine including Na and K follow circadian patterns^4, 25–27^. The molecular clock in the kidney may directly regulate renal functions^9^. *NHE3* coding Na^+^/H^+^ exchanger, *aENaC* coding alpha subunit of epithelial sodium channel, and *Aqp2* coding water channel are reported as CCGs^28–31^. Nikolaeva *et al*. performed comprehensive gene expression analysis and found that 277 transcripts showed circadian oscillation in kidneys, whereas the cycling transcripts decreased to 174 in *Clock* mutant mice^26^.

Although it has been reported that the expression of clock genes showed apparent daily rhythms, functional variations of regional clocks in the kidney have not yet been fully understood. Here we described molecular clock oscillation in the IM and the cortex, and diurnal rhythms of both osmotic pressure in the IM and cortico-medullary osmotic pressure gradient. Furthermore, we studied diurnally expression patterns of genes such as vasopressin receptors, urea transporters, and water channels in mice. Finally, we examined day-night variations of cortico-medullary osmotic pressure gradient in *Bmal1* deficient mouse kidneys.

## Results

### Localization of PER2::Luc activity in slice cultured mouse kidney

**Figure 1:**
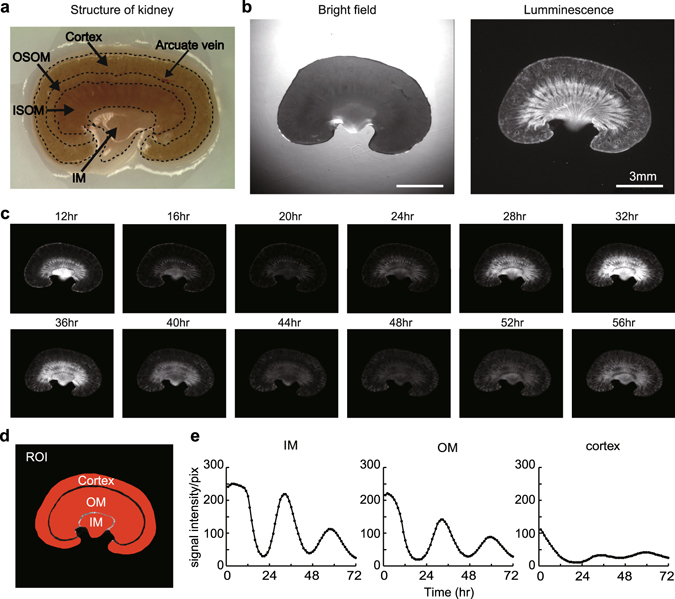
Localization of PER2::Luc activity in kidney. (**a**) Anatomical structure of mouse kidney. (**b**) PER2::Luc activity in slice cultured kidney. A bright field image (left) and a luminescence image (right) are displayed. White horizontal bar indicates 3 mm. (**c**) Time series observation of the bioluminescence in a slice cultured kidney. We set 0hr when we started observations. **(d**,**e**) Quantitative analysis of regions of interest (ROIs) in the kidney slice. Set ROIs are shown in (**d**). Circadian rhythmic signal intensities were observed in the cortex, OM, and IM (**e**). OM, outer medulla; IM, inner medulla; OSOM, Outer stripe of outer medulla; ISOM, Inner stripe of outer medulla.

By high-sensitivity charge-coupled device (CCD) camera-based macroscopic imaging systems with PERIOD2::LUCIFERASE (PER2::Luc) knock-in mouse which express firefly luciferase fused PER2 protein^11^, we studied the localization of the PER2::Luc activity in the kidney (Fig. 1a–c). We found strong signals in the medulla, along with renal tubule. PER2::Luc activity was also observed in the cortex and small spotted signals comparable to glomerulus were found (Fig. 1b,c). Quantitative analysis by setting regions of interests (ROIs) in the IM, OM and cortex revealed bioluminescence in these areas clearly circadian oscillated lasting at least three days (Fig. 1d,e, Supplementary Video S1). Noticeably, the bioluminescence rhythm in the IM and OM showed higher amplitude than the cortex (Fig. 1e).

### Diurnal rhythms of the osmotic pressure and osmotic pressure gradient

**Figure 2:**
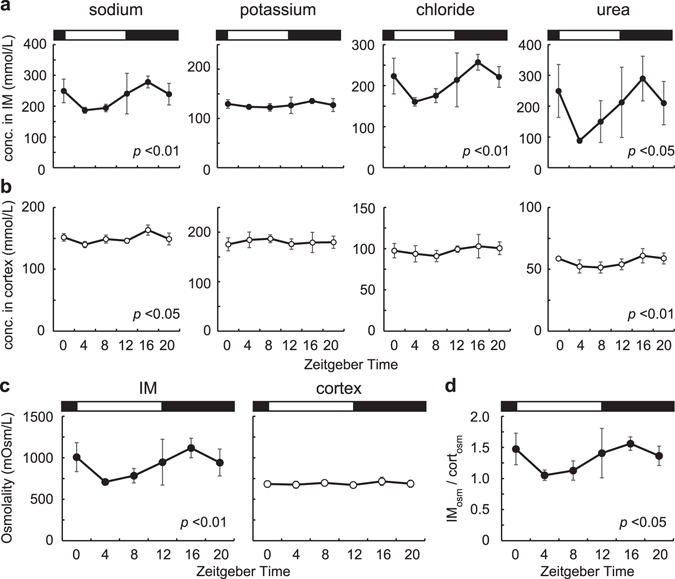
Diurnal variations of electrolytes, urea concentrations, and osmotic pressure in rat kidney. **(a–b)** Diurnal variations of electrolytes and urea concentrations in the IM (**a**) and cortex (**b**). From left to right, diurnal variations of sodium, potassium, chloride, and urea concentrations are shown. (**c**) Osmotic pressure in the IM (left) and cortex of the rat kidney (right). (**d**) IM-cortex osmotic pressure ratio (IM_osm_/cort_osm_) representing osmotic pressure gradient. Mean values ± standard deviations (S.D.) (n = 4). White and black rectangles in graphs indicate the light phase and the dark phase, respectively.

Next, we collected rat kidneys at Zeitgeber time (ZT) 0, 4, 8, 12, 16, and 20 where ZT12 is the time of light off. We measured electrolytes and urea concentrations, and calculated osmotic pressure in the IM and cortex. We confirmed that Na, Cl, and urea concentrations were higher, whereas K was lower in the IM than in the cortex (Fig. 2a,b), as reported previously^32, 33^. In the IM, significant diurnal rhythms were detected in Na (p < 0.01), Cl (p < 0.01), and urea concentrations (p < 0.05), but not in K concentrations (p = 0.750) (Fig. 2a). In the cortex, Na (p < 0.05) and urea (p < 0.01), but not K (p = 1.00) and Cl concentrations (p = 0.142) showed diurnal rhythms, while amplitudes of Na and urea rhythms in the cortex were lower than those in the IM (Fig. 2a,b). Significant diurnal rhythm of osmotic pressure peaking at night was detected in the IM (p < 0.01), but not in the cortex (p = 1.00) (Fig. 2c). Furthermore, the ratio that IM osmotic pressure divided by cortex osmotic pressure (IM_osm_/cort_osm_) showed significant diurnal rhythm (p < 0.05) (Fig. 2d). This suggests that cortico-medullary osmotic pressure gradient changes diurnally depending on osmotic pressure rhythm in the IM.

### Diurnal rhythms of gene expressions in the IM and cortex of the kidney

**Figure 3:**
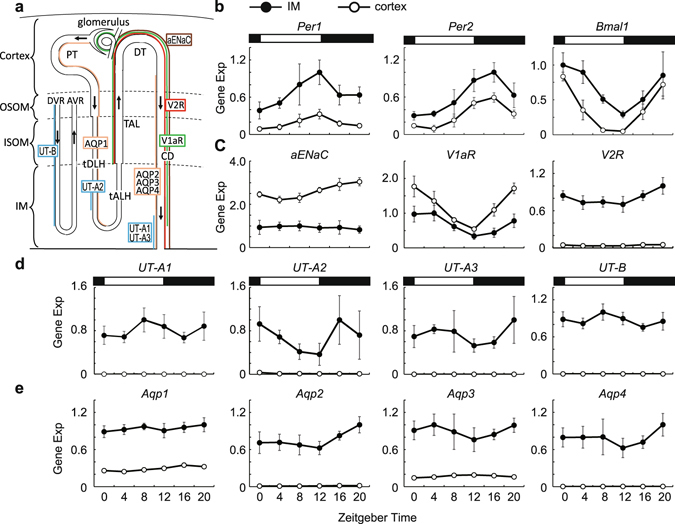
Clock genes and genes contributing high osmotic pressure gradient and water re-absorption in mouse kidney. (**a**) Diagrammatic representation of the localization of analized genes in the long-looped nephron and vasa recta. *Aqp1* is expressed in proximal tubules (PT) and thin descending limbs of Henle’s loop (tDLH) (orange). *Aqp2*, *Aqp3*, and *Aqp4* are expressed in the collecting ducts (CD) (orange). *V1aR* is expressed the thick ascending limbs (TAL) of Henle’s loop, distal tubule (DT), and the CD except for deeper third of the inner medullary CD, and glomerulus (green). V2R is expressed in the TAL, DT, and the CD (red). *UT-A1* and *UT-A3* are expressed in the deepest part of the inner medullary CD (blue). *UT-A2* is expressed in a limited portion of tDLH (blue). *UT-B* is expressed in the descending vasa recta (DVR) throughout the medulla (blue). *aENaC* is expressed the DT and the CD (brown). The other abbreviations used are: OSOM, Outer stripe of outer medulla; ISOM, Inner stripe of outer medulla; IM, Inner medulla; AVR, ascending vasa recta; tALH, thin ascending limb of Henle. (**b**–**e**) Diurnal variations of *Per1*, *Per2*, *Bmal1* (**b**), *aEnac*, vasopressin receptors (**c**), urea transporters (**d**), and water channels (**e**). Each gene expressions in the IM (•) and cortex (⚬) are shown. Data were normalized to 18S ribosomal RNA (mean values ± S.D, n = 6). White and black rectangles in graphs indicate the light phase and the dark phase, respectively.

Then, we analyzed diurnal expression patterns of genes contributing high osmotic pressure in the IM and re-absorption water in the IM and cortex of mouse kidneys by quantitative PCR (qPCR) analysis (Fig. 3a). In the IM, we confirmed clock genes, *Per1* (p < 0.001), *Per2* (p < 0.001) and *Bmal1* (p < 0.001) showed diurnal rhythms with known phase relationships (Fig. 3b). *aENaC* expressions in the IM were lower through a day comparing to that in the cortex, and showed no significant rhythm (p = 1.00) (Fig. 3c). Vasopressin receptors, *V1aR* (p < 0.001) and *V2R* (p < 0.001), showed diurnal rhythms in the IM (Fig. 3c). According to urea transporters (*UT-A1*, *UT-A2*, *UT-A3* and *UT-B*), only *UT-A2* showed a diurnal rhythm (p < 0.01) in the IM (Fig. 3d). Among the analyzed four water channels, we detected diurnal rhythm of *Aqp2* in the IM (p < 0.01) (Fig. 3e).

In the cortex, *Per1* (p < 0.001), *Per2* (p < 0.001) and *Bmal1* (p < 0.001) showed diurnal rhythms (Fig. 3b). We detected significant diurnal rhythms in *aENaC* (p < 0.001), *VIaR* (p < 0.001) and *V2R* (p < 0.001) in the cortex (Fig. 3c **)**. Comparing to expression levels in the IM, *V1aR* was higher whereas *V2R* was lower in the cortex (Fig. 3c). *UT-A1*, *UT-A2*, *UT-A3*, and *UT-B* were hardly detected in the cortex (Fig. 3d). Significant diurnal rhythms in *Aqp1* (p < 0.001), *Aqp2* (p < 0.001), and *Aqp3* (p < 0.001) were detected in the cortex, although expression levels of these genes were relatively low (Fig. 3e). *Aqp4* expression was hardly detected in the cortex (Fig. 3e).

### Impaired inner medullary rhythms in *Bmal1* deficient mice

**Figure 4:**
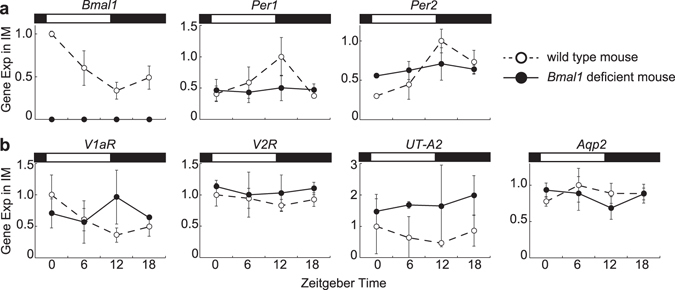
Diurnal variations of clock gene expressions (**a**) and diurnal variation of *UT-A2*, *Aqp2*, *V1aR*, and *V2R* (**b**) in the IM. Data obtained from *Bmal1* deficient mouse (**•**) and age matched wild type mouse (◦) are shown. Data were normalized to 18S ribosomal RNA (mean values ± S.D, n = 3). White and black rectangles in graphs indicate light phase and dark phase, respectively.

To analyze the necessity of molecular clock in diurnal variations of osmotic pressure gradient, we studied osmotic pressure, electrolytes and urea concentrations in systemic *Bmal1* deficient mice which completely lack a functional clock^34^. As shown in Fig. 4a, all three clock genes (*Bmal1*, *Per1* and *Per2*) diurnally oscillated as expected in wild type littermates (p < 0.01, p < 0.05 and p < 0.001, respectively). In contrast, *Per1* and *Per2* expressions showed no significant rhythms (p = 1.00, p = 0.093, respectively), and *Bmal1* expression was hardly detected in *Bmal1* deficient mice (Fig. 4a). Moreover, *UT-A2*, *Aqp2*, *V1aR*, and *V2R* showed no significant rhythms in *Bmal1* deficient mice (Fig. 4b).

**Figure 5:**
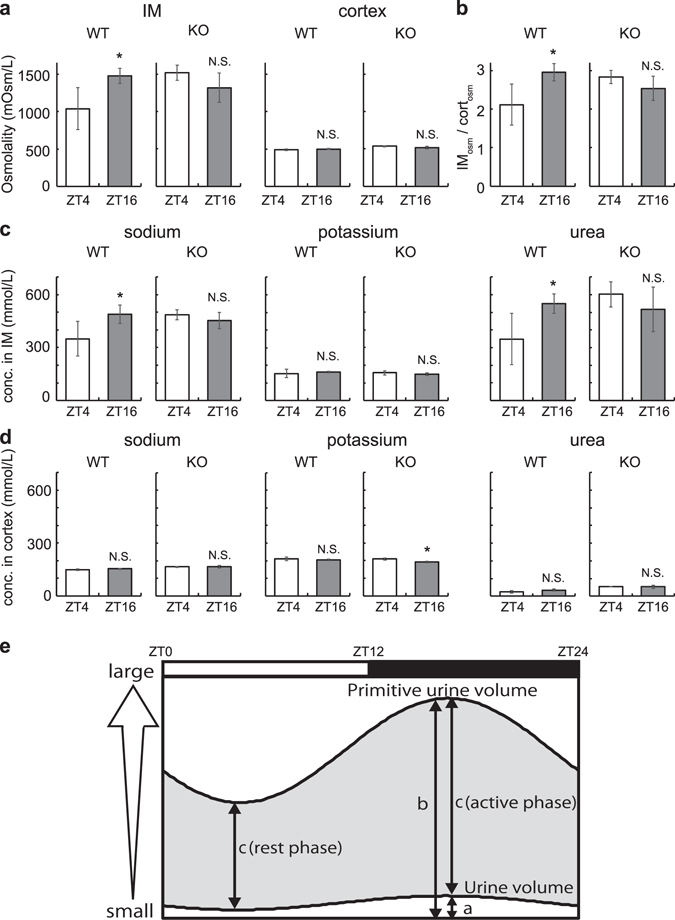
Day-night variations of osmotic pressure, electrolytes, and urea concentrations in wild type and *Bmal1* deficient mouse kidney. (**a**) Osmotic pressure in the IM (left) and cortex of the mouse kidney (right). (**b**) IM-cortex osmotic pressure ratio (IM_osm_/cort_osm_) representing osmotic pressure gradient. (**c**,**d**) Day-night difference of electrolytes and urea concentrations in the IM (**c**) and cortex (**d**). From left to right, day-night difference of sodium, potassium, chloride, and urea concentrations are shown. We used pooled IM and cortex samples from three mice as one sample for measurement and performed four independent experiments. Values are shown as mean values ± S.D. *p < 0.05 versus ZT4. N.S., not significant. (**e**) Schematic view of primitive urine volume and urine volume. *a* and *b* denotes urine volume and primitive urine volume, respectively. *c* denotes water re-absorption volume that equals to difference between primitive urine volume and urine volume (gray shade). Both *a* and GFR (reflecting in *b*) are larger at night^23^. As *b* is nearly a hundred-times larger than *a* ^50^, circadian variation of *b* is mainly reflected in the circadian variation of *c*. Therefore, (**c**) (active phase) is higher than *c* (rest phase). White and black rectangles in schema indicate the light phase and the dark phase, respectively.

In wild type mice, osmotic pressure in the IM at ZT16 was 1.42 times higher than that at ZT4 (p = 0.027), and IM_osm_/cort_osm_ value reflecting osmotic pressure gradient at ZT16 was 1.40 times higher than that at ZT4 (p = 0.027) (Fig. 5a,b). These results are similar to day-night variations in rat kidneys (Fig. 2). Adding to these, we found Na and urea in the IM were also higher in ZT16 than ZT4 (p = 0.047 and p = 0.041, respectively) (Fig. 5c). In *Bmal1* deficient mice, however, osmotic pressure in the IM at ZT16 was not different from that at ZT4 (p = 0.118) **(**Fig. 5a). Similarly, IM_osm_/cort_osm_ value at ZT16 was not different from that at ZT4 (p = 0.143) (Fig. 5b). Na and urea concentrations also showed similar patterns in *Bmal1* deficient mice (Fig. 5c). Any day-night variations in osmotic pressure and electrolytes except for K in the cortex was not observed in either wild type or *Bma11* deficient mice (Fig. 5a,d).

## Discussion

In this study, we revealed the tissue-autonomous circadian clock oscillation in cultured kidneys using bioluminescence macroscopic imaging devices. Our finding that strong and robust circadian oscillation of the bioluminescence in the medulla highlights molecular clock functions in medullas, including a thin descending and ascending limb of loop of Henle and collecting duct (Fig. 1, Supplementary Video S1). Local clock functions in the kidney have been approached by transcriptomic study using laser micro-dissected samples or physiological analysis using several kinds of conditional KO mice^35, 36^. However, most studies have analyzed molecular clocks on the whole kidney level or cortex, whereas few studies have focused on the molecular clock in functions of the medulla^26, 28, 31^. In this study, by performing real-time bioluminescence imaging analysis at the organ level, we succeeded not only in showing regional tissue-autonomous molecular clocks oscillating in the kidney, but also to highlight local clock functions in the IM.

Urine volume displays a circadian rhythm, but the whole mechanism remains unclear. Micturition volume is influenced by bladder capacity which is under circadian control via *Cox43* ^22^. Urine production is depended on several parameters including blood pressure, the renal blood flows (RBF), the glomerular filtration rate (GFR), blood vasopressin levels, the cortico-medullary osmotic pressures gradient^4, 37^. Blood pressure exhibits a robust circadian rhythm peaking at active phase^38^, and urine volume also has circadian rhythm peaking at active phase^20–22^. Moreover, both *Bmal1* deficient mice and *Cry1*, *Cry2* double knock out mice (*Cry*-null mice) that abolish clock gene regulation in whole body completely lose the circadian variation in blood pressure^38, 39^, and *Cry*-null mice also lose urination rhythm^39^. These results suggest that blood pressure rhythmicity may affect the urine volume rhythmicity. Cortico-medullary osmotic pressure gradient also affect urine volume via water reabsorption. In this study, we revealed, for the first time, that the osmotic pressure in the IM and the osmotic pressure gradient is higher at night than those during the day in rats (Fig. 2c). Supporting this, we observed similar results in mice (Fig. 5a). Our data indicate that diurnal rhythms of cortico-medullary osmotic pressure gradients also may contribute to urine volume rhythmicity. Furthermore, day-night variations of osmotic pressure in the IM and the osmotic pressure gradient was disrupted in *Bmal1* deficient mice (Fig. 5a,b). Together with strong circadian clock oscillation in the IM observed by macro-imaging analysis, these data suggest that the medullary clock plays an important role in generating cortico-medullary osmotic pressure gradient rhythm.

Urea is considered a key player for generating high osmotic pressure in the IM^16^. In this study, we found urea concentrations in the IM showed diurnal rhythm peaking at night-time (Fig. 2a). Urea transporters, *UT-A1*, *UT-A2*, and *UT-A3* are transcribed from the same gene, *Slc14a2*. *UT-A1* and *UT-A3* are driven by UT-A alpha promoter whereas *UT-A2* is driven by UT-A beta promoter^40^. qPCR analysis revealed that *UT-A2* showed diurnal rhythm in the IM peaking at night **(**Fig. 3d). By facilitating urea recycling between the inner medulla collecting duct and thin descending limb segments, UT-A2 is thought to support for maintaining hypertonic medulla that provides the driving force for water re-absorption from the collecting ducts^41^. Rhythmic expression of *UT-A2* may contribute for generating urea accumulation rhythm in the IM. Note that UT-A1 and UT-A3 mainly functions for urea accumulation and UT-A2 contribution is restricted in the IM^42^. Although no significant diurnal variations were detected in *UT-A1* and *UT-A3* expressions (Fig. 3d), these transporters may play roles for urea accumulation rhythm through vasopressin-V2R pathway. Vasopressin induces phosphorylation of UT-A1 and UT-A3 via V2R, increases UT-A1 and UT-A3 expression in cellular membrane, and increases urea permeability in the terminal of the inner medullary collecting duct^43, 44^. Noteworthy, peak phase of *V2R* expression in the IM came at night as the same phase as urea concentrations rhythm in the IM (Figs 2a and 3c).

In humans, some studies showed that vasopressin concentrations increase at night (rest phase) and it results in urine volume decrease and urine concentration increase at rest phase^45^. The circadian rhythm of blood vasopressin levels is under discussion. Although some studies reported diurnal rhythm of serum vasopressin level peaking at late resting phases in humans and rats^45–47^, others observed no rhythmicity^48, 49^. This inconsistency probably occurred from low concentrations of vasopressin levels^49^. Noticeably, vasopressin receptors, *V1aR* and *V2R*, are revealed to show circadian rhythm^31^. We also observed the same results (Fig. 3c). As V2R mediates the anti-diuretic effect of vasopressin in the kidney, this suggests that circadian rhythmic functions via vasopressin signalling may be achieved by rhythmic expression of vasopressin receptors.

AQP2 play roles in water re-absorption at cellular membrane, and vasopressin signal via V2R regulates membrane trafficking of AQP2. We found rhythmic expressions of *V2R* and *Aqp2* became high during night in the IM (Fig. 3c,e). Although water re-absorption is mainly regulated in the IM, about two thirds of re-absorption occurs in proximal tubule which are located in the cortex^50^. In the cortex, though amplitude was low, we found the rhythmic expression of *Aqp1* which plays an important role in water re-absorption there. Like *V2R* and *Aqp2* in the IM, *Aqp1* expression peak was night. Gathering all data observed in this study including osmotic pressures and urea concentrations in the IM, water re-absorption rate is suggested to be high at night (active phase) and low during the day (rest phase).

Diurnal rhythm of water re-absorption peaking at the active phase is also suggested from diurnal rhythms of urine volume and GFR^23^. Since the volume of primitive urine is nearly a hundred-times larger than excreted urine volume^50^, it is consistent with the in-phase circadian rhythms of primitive urine and water re-absorption as shown in Fig. 5e. The mechanism of urine concentration is tightly regulated because even slight changes in primitive urine re-absorption can lead significant effects on body fluid homeostasis. The circadian clock is likely to regulate osmotic pressure gradient actively for dealing with increasing water re-absorption at active phases effectively.

In conclusion, we identified robust regional of circadian clock oscillation in the medulla of kidneys by real-time bioluminescence imaging using macroscopic observation devices, which enabled us to evaluate the circadian clock rhythmicity in the whole kidney level. This approach highlights importance of IM circadian clock. We found that osmotic pressure in the IM showed diurnal rhythms, but not in the cortex. By qPCR analysis, as well as clock genes, vasopressin receptors (*V1aR*, *V2R*), water channel (*Aqp2*), and urea transporter (*UT-A2*) expression levels were identified to show diurnal rhythms in the IM. We found that osmotic pressure gradient was high at night (active phase) but not in *Bmal1* deficient mice. These findings suggest that the circadian clock affected the generation of cortico-medullary osmotic pressure gradient rhythm via, at least in part, molecular rhythms of urea transporters, water channels, and vasopressin receptors.

## Methods

### Ethics Statement

All experiments were approved by the Experimental Animals Committee, Kyoto Prefectural University of Medicine, and were performed in accordance with the institutional guidelines and Guidelines for Proper Conduct of Animal Experiments by Science Council of Japan.

### Animals

BothPER2::Luc K.I mice^11^ and *Bmal1* deficient mice were kept under 12-hour light and 12-hour dark (LD) conditions in our facility^34^. Genotyping of *Bmal1* deficient mice was performed using PCR^34^. Both Slc-Wistar rats (10 weeks old) and C57BL/6 J mice (10 weeks old) were purchased from Shimizu Experimental Materials (Kyoto, Japan), and maintained at least 2 weeks under LD conditions in our facility. Food and water were available *ad libitum*.

### Slice culture and macroscopic bioluminescence imaging

A kidney was collected from PER2::Luc mouse (12 weeks old, male), and sliced using a linear slicer (250 μm-thick) (Dosaka EM, Osaka, Japan). The slice was placed directly into the cell culture inserts (PICM ORG50, Millipore, MA, United States) in dishes with 1.2 ml of the culture medium containing phenol red-free DMEM (Nacalai Tesque, Kyoto, Japan), 10 mM HEPES (Nacalai Tesque, Kyoto, Japan), 1% Glutamax (Life Technologies, Carlsbad, CA, United States), 1 mM Sodium pyruvate (Nacalai Tesque, Kyoto, Japan), 2% B-27 supplement (Life Technologies, Carlsbad, CA, United States), 200 μM beetle luciferin (Promega, Madison, WI, USA), 100 units/ml penicillin, and 100 mg/ml streptomycin (Nacalai Tesque, Kyoto, Japan). The culture dish was set in the high-sensitivity charge-coupled device (CCD) camera-based microscopic imaging systems (Olympus, Tokyo, Japan; ATTO, Tokyo, Japan) and images were taken every hour. Further analysis was performed using AquaCosmos software (Hamamatsu Photonics, Hamamatsu, Japan).

### Measurement of osmotic pressure

Rat kidneys were processed as Schimidt-Nielsen *et al*. described^51^ with little modifications^52^. Briefly, rats (12 weeks old, male) were anesthetized with isoflurane, and sacrificed for collecting both left and right kidneys every 4 hours throughout a day (n = 4 in each time point). Kidneys were dissected, and white color region were collected as IM. To collect the cortex, arcuate artery, arcuate vein, and the color difference between the cortex and OM were used as landmarks. The cortex and IM were placed on the filter paper to remove residual urine and blood, were dried at 60 °C for 6 hours in plastic tubes. After adding distilled water, tubes were tightly sealed and placed at 90 °C for 3 min, and then stored at 4 °C for 24 hours for diffusion. Samples were centrifuged for 1 min. at 8000 × g, and supernatant was used for measurement. We measured Na, K, Cl, and urea concentrations by usingFDC7000V (Fujifilm, Tokyo, Japan). The osmotic pressure was estimated as the sum of the urea concentration and two times the sum of Na and K concentration. We multiplied the obtained values by distilled water volume/water content, where water content was calculated by subtracting dry weight from wet weight of the collected IM or cortex. We used TG204 DeltaRange, d = 0.1 mg (METTLER TOLEDO, Greifensee, Switzerland) for measuring rat kidney weight.

Similar to rats, mouse kidneys were divided into IM and Cortex, pooled into plastic tubes separately, and dried as described above. Since mouse kidneys are smaller than rat, the kidneys collecting from three mice were pooled as one sample at each time point and we performed four independent studies. The osmotic pressure was measured using 5600 Vaper Pressure Osmometer (WESCOR, Logan, UT, United States), and electrolytes were measured using FDC7000V (Fujifilm, Tokyo, Japan). We used AUW120D, d = 0.01 mg (SHIMADZU CORPORATION, Kyoto, Japan) for measuring mouse kidney weight.

### RNA extraction and quantitative PCR

Mice kidneys were collected at ZT0, 4, 8, 12, 16, and 20 (n = 6). To asses *Bmal1* deficient effect, we collected kidneys from *Bmal1* deficient mice and wild type mice at ZT0, 6, 12, and 18 (n = 3). After collection, the IM and cortex were divided, frozen by liquid nitrogen, and stored in an −80 °C deep freezer until RNA isolations. Total RNA extraction and cDNA synthesis were performed as described previously^53^. The amplification protocol comprised an initial incubation at 95 °C for 10 min and 40 cycles of 95 °C for 15 sec and 60 °C for 1 min, followed by melting curve analysis. Gene expression levels were calculated from relative standard curves and normalized to 18s ribosomal RNA. Primer sequences were listed in Supplementary Table S1.

### Statistical analysis

For detecting rhythmicity, cycling analysis was performed by JTK_cycle^54^ from Metacycle package^55^ with options of minper = 22 and maxper = 26. To compare two groups (*Bmal1* deficient mice and their wild type littermates) in Fig. 5, statistical analysis was performed by Two-tailed Student’s T-test. The P-value < 0.05 was considered statistically significant. The P-values indicated was determined by JTK_cycle otherwise noted.

## Electronic supplementary material

Supplementary Video S1

Supplementary Information
