# Supplementary material for: Robust circadian clock oscillation and osmotic rhythms in inner medulla reflecting cortico-medullary osmotic gradient rhythm in rodent kidney
Source: Sci Rep. 2017 Aug 4;7:7306. doi: 10.1038/s41598-017-07767-8 (PMC5544761; doi:10.1038/s41598-017-07767-8)
Supplement: Supplementary file 2 — Supplementary Information [file 41598_2017_7767_MOESM2_ESM.pdf]

Supplementary information:

Robust circadian clock oscillation and osmotic rhythms in inner medulla reflecting cortico-medullary osmotic gradient rhythm in rodent kidney

Masayuki Hara, MD[1,2], Yoichi Minami, PhD[1], Munehiro Ohashi, MD[1], Yoshiki Tsuchiya, PhD[1], Tetsuro Kusaba, MD, PhD[2], Keiichi Tamagaki,MD, PhD[2], Nobuya Koike, PhD[1], Yasuhiro Umemura, PhD[1], Hitoshi Inokawa, PhD[1], and Kazuhiro Yagita, MD, PhD[1,*]

1. Department of Physiology and Systems Bioscience, and 2. Department of Nephrology, Graduate School of Medical Science, Kyoto Prefectural University of Medicine, Kyoto, Japan.

Supplementary Video S1. The real-time bioluminescence imaging of the kidney slice using CCD camera-based macroscopic imaging system related to Fig1.

A clear circadian rhythm of bioluminescence was observed in cortex and medulla, and signals in medulla is stlong along with renal tubule. The time scalae shown on the higher left corner indicats "hours: minutes: seconds". Yellow horizontal bar indicates 4mm.

Supplementary Table S1: Primer sequences used for qPCR.

Primer sequences we used for qPCR are listed. PCR primers sequences were followed acording to previous papers except *Per1*, *Per2*, *Bmal1*. *Rn18s* from Hosokawa et al.[1], *V1aR* from Zhao et al.[2], *V2R* from Starremans et al.[3], *Aqp1, Aqp2, Aqp4, UT-A1, UT-A2, UT-A3, UT-B* from Klein et al.[4], *Aqp3* from McReynolds et al.[5], and *aENaC* from Malsure et al[6]. Primer sequences of *Per1*, *Per2*, and *Arntl* were designed for this study.

Supplementary Table S1: Primer sequences used for qPCR

Official name (Gene ID)	Primer name	Sequence	Reference
Rn18s (19791)	18s-F	CGCCGCTAGAGGTGAAATTC	(1)
	18s-R	CGAACCTCCGACTTTCGTTCT	(1)
Avpr1a (54140)	V1aR-F	TTCATCGTCCAGATGTGGTCAGTC	(2)
	V1aR-R	GCTCTGGACACAATCTTGTAGGAG	(2)
Avpr2 (12000)	V2R-F	ATGATCCTGGTGTCTACCACGT	(3)
	V2R-R	ACTAACAGCGGGTCTCGGTC	(3)
Aqp1 (11826)	Aqp1-F	CTCCCTAGTCGACAATTCAC	(4)
	Aqp1-R	ACAGTACCAGCTGCAGAGTG	(4)
Aqp2 (11827)	Aqp2-F	CTGGCTGTCAATGCTCTCCAC	(4)
	Aqp2-R	TTGTCACTGCGGCGCTCATC	(4)
Aqp3 (11828)	Aqp3-F	GCCCTCCAGAATTTCTATGAACTCT	(5)
	Aqp3-R	TTTGCTATCCTACCTTGGCTTAAAG	(5)
Aqp4 (11829)	Aqp4-F	GAGTCACCACGGTTCATGGA	(4)
	Aqp4-R	CGTTTGGAATCACAGCTGGC	(4)
Scnn1a (20276)	αENaC-F	GCACCCTTAATCCTTACAGATACACTG	(6)
	αENaC-R	CAAAAAGCGTCTGTTCCGTG	(6)
Slc14a2 (27411)	UT-A1-F	GACAGTGAGACGCAGTGAAG	(4)
	UT-A1-R	ACGGTCTCAGAGCTCTCTTC	(4)
Slc14a2 (27411)	UT-A2-F	TTTCTCCAGTCCTATCTGAG	(4)
	UT-A2-R	ACGGTCTCAG？AGCTCTCTTC	(4)
Slc14a2 (27411)	UT-A3-F	GACAGTGAGACGCAGTGAAG	(4)
	UT-A3-R	AGAGTGGAGGCCACACGGAT	(4)
Slc14a1 (108052)	UT-B-F	TCTTCTCAAACAAGGGCGAC	(4)
	UT-B-R	TTGCTGAGCACGGAGCTCAA	(4)
Per1 (18626)	Per1-F	CCCAGCTTTACCTGCAGAAG	
	Per1-R	ATGGTCGAAAGGAAGCCTCT	
Per2 (18627)	Per2-F	CAGCACGCTGGCAACCTTGAAGTAT	
	Per2-R	CAGGGCTGGCTCTCACTGGACATTA	
Arntl (11865)	Bmal1-F	TAAACTCACCGTGCTAAGGATG	
	Bmal1-R	TGGCTTGTAGTTTGCTTCTGTG	

Supplementary Reference

1. Hosokawa, T. *et al.* Robust Circadian Rhythm and Parathyroid Hormone-Induced Resetting during Hypertrophic Differentiation in ATDC5 Chondroprogenitor Cells. *Acta histochemica et cytochemica*. **48**, 165-171 (2015).
2. Zhao, D., Bankir, L., Qian, L., Yang, D. & Yang, B. Urea and urine concentrating ability in mice lacking AQP1 and AQP3. *Am J Physiol Renal Physiol*. **291**, F429-438 (2006).
3. Starremans, P. G. *et al.* A mouse model for polycystic kidney disease through a somatic in-frame deletion in the 5' end of Pkd1. *Kidney international*. **73**, 1394-1405 (2008).
4. Klein, J. D., Sands, J. M., Qian, L., Wang, X. & Yang, B. Upregulation of urea transporter UT-A2 and water channels AQP2 and AQP3 in mice lacking urea transporter UT-B. *J Am Soc Nephrol*. **15**, 1161-1167 (2004).
5. McReynolds, M. R., Taylor-Garcia, K. M., Greer, K. A., Hoying, J. B. & Brooks, H. L. Renal medullary gene expression in aquaporin-1 null mice. *Am J Physiol Renal Physiol*. **288**, F315-321 (2005).
6. Malsure, S. *et al.* Colon-specific deletion of epithelial sodium channel causes sodium loss and aldosterone resistance. *J Am Soc Nephrol*. **25**, 1453-1464 (2014).
